# Enhanced Phosphorylation of Bax and Its Translocation into Mitochondria in the Brains of Individuals Affiliated with Alzheimer’s Disease

**DOI:** 10.2174/1874205X01711010048

**Published:** 2017-11-16

**Authors:** L.E. Henderson, M.A. Abdelmegeed, S.H. Yoo, S.G. Rhee, X. Zhu, M.A. Smith, R.Q. Nguyen, G. Perry, B.J. Song

**Affiliations:** 1 Laboratory of Membrane Biochemistry and Biophysics, National Institute on Alcohol Abuse and Alcoholism, Bethesda, Maryland 20892-9410, USA; 2 Division of Life and Pharmaceutical Sciences, Ewha Womans University, Seoul, Korea; 3 Department of Pathology, Case Western Reserve University, Cleveland, Ohio 44106, USA; 4 Department of Biology, College of Sciences, University of Texas at San Antonio, San Antonio, TX, USA

**Keywords:** Alzheimer’s disease, p38 kinase, Bax phosphorylation, Mitochondrial translocation, Apoptosis, Neurodegeneration

## Abstract

**Background:**

Despite increased neuronal death, senile plaques, and neurofibrillary tangles observed in patients suffering from Alzheimer’s disease (AD), the detailed mechanism of cell death in AD is still poorly understood.

**Method:**

We hypothesized that p38 kinase activates and then phosphorylates Bax, leading to its translocation to mitochondria in AD brains compared to controls. The aim of this study was to investigate the role of p38 kinase in phosphorylation and sub-cellular localization of pro-apoptotic Bax in the frontal cortex of the brains from AD and control subjects. Increased oxidative stress in AD individuals compared to control was evaluated by measuring the levels of carbonylated proteins and oxidized peroxiredoxin, an antioxidant enzyme. The relative amounts of p38 kinase and phospho-Bax in mitochondria in AD brains and controls were determined by immunoblot analysis using the respective antibody against each protein following immunoprecipitation.

**Results:**

Our results showed that the levels of oxidized peroxiredoxin-SO_3_ and carbonylated proteins are significantly elevated in AD brains compared to controls, demonstrating the increased oxidative stress.

**Conclusion:**

The amount of phospho-p38 kinase is increased in AD brains and the activated p38 kinase appears to phosphorylate Thr residue(s) of Bax, which leads to its mitochondrial translocation, contributing to apoptosis and ultimately, neurodegeneration.

## 1 INTRODUCTION

Alzheimer’s disease (AD) is a common age-related neurodegenerative disorder, which is initially marked by the loss of recent memory and leads to further loss of cognition. Three main pathological hallmarks of AD – senile plaques neurofibrillary tangles, and inappropriate activation of neuronal loss (apoptosis) – all aid in the diagnosis and progression of this neurodegenerative disease. Many reports have shown neuronal loss through the decrease in volume and density of the brains of individuals with AD [1, 2]. This decrease in neuronal cells is a major cause of the neurodegeneration associated with AD [3], and has been shown to parallel the severity of the disease: increased neuronal loss occurs with the progression of AD stages, even including marked decreases in neuronal number in early stages of AD [4].

Like neuronal loss, oxidative stress plays a critical role in the early stages of AD, leading to the accrual of neurodegeneration [2, 5]. It has been shown that oxidative stress with overtly increased amounts of reactive oxygen species (ROS) drives the cell towards cell death, which ultimately contributes to neurodegeneration [6-9]. ROS lead to cell death through the accumulation of oxidized proteins, lipids and DNA while activating the members of the mitogen-activated protein kinase (MAPK) proteins [10, 11].

The MAPK family members are known to regulate cell death or proliferation depending on the time, duration, and magnitude of their activation as well as cellular context. In general, activation (phosphorylation) of c-Jun N-terminal kinase and p38 kinase (p38K) is associated with cell death, while activated extracellular signal-regulated kinase is related with cell growth and proliferation [12]. Previous research revealed that the number of apoptotic, but not necrotic, cells is significantly increased in AD specimens compared to control [13-15]; however, the mechanism through which this MAPK-mediated apoptosis occurs is relatively unknown. Furthermore, to our knowledge, the sub-cellular location of pro-apoptotic Bax and the mechanism for its translocation to mitochondria have not been studied in AD brains, although we have previously demonstrated that activated p38K phosphorylates Bax before it is translocated to mitochondria to initiate mitochondria-dependent apoptosis in cultured hepatoma cells [16]. Based on this well-established information, we hypothesized that p38K is activated and then phosphorylates Bax, leading to its translocation to mitochondria in AD brains compared to controls. To answer this hypothesis and elucidate a possible mechanism of neuronal apoptosis, this study was aimed to evaluate the role of oxidative stress in the activation of p38K and subsequent phosphorylation and mitochondrial translocation of Bax in the frontal cortex tissue of human AD and control subjects.

## 2 EXPERIMENTAL PROCEDURES

### 2.1 Tissue Specimens

The tissue specimens used in this study were brain tissues from the frontal cortex of individuals diagnosed with AD (n=4) and control (n=3) individuals and an additional set consisting of 2 other AD individuals and 2 other control specimens (all from the University Memory Center, Case Western Reserve University, Cleveland, OH, USA). All experiments with human subjects were conducted in accordance with the Declaration of Helsinki. The demographic data of each specimen is summarized in Table **1**. Each tissue from different individuals was homogenized in STE buffer (containing 250 mM sucrose, 50 mM Tris-Cl, pH 7.5, and 1 mM EDTA with protease and phosphatase inhibitor cocktails) by following the published method [17] with slight modifications [18]. Whole tissue homogenates were centrifuged at 600 x g at 4°C for 5 min and the supernatant was carefully removed. This supernatant was then centrifuged at 9,000 x g at 4°C for 20 min. The supernatant, of this second spin, was kept as the cytosolic fractions and used for immunoblot analysis. The remaining pellets were washed with ice-cold STE buffer three times to remove the contaminating cytosolic proteins and used as the crude mitochondrial fractions, as previously described [16, 18]. Protein concentrations, of both sets of fractions, were determined using the Bio-Rad protein assay kit, as previously described [16].

### 2.2 Antibodies

All specific antibodies against p38K, phospho-p38K, phospho-Thr-Pro, or phospho-Ser-Pro were purchased from Cell Signaling Technology, Inc. (Danvers, MA, USA). Anti-Bax 2D2, anti-Bax 6A7, anti-β-actin, and anti-ATP synthase β-subunit were obtained from Sigma Chemical (St. Louis, MO, USA). Specific antibodies to peroxiredoxin (Prx)-SO_3_ and Prx II were procured from LabFrontier Co. Ltd. (Seoul, Korea). Secondary antibodies, conjugated with horseradish peroxidase, were obtained from Santa Cruz Biotechnology, Inc. (Santa Cruz, CA, USA).

### 2.3 Measurement of Protein Carbonylation

The OxyELISA Oxidized Protein Quantitation Kit (Millipore, Bedford, MA, USA) was used to measure the amount of protein carbonylation as an indicator of protein oxidation/oxidative stress in a given sample. The protein homogenates (100 μL volume at 10 μg protein/mL, run in duplicate), prepared by following the manufacturer’s instructions, along with 100 μL of each standard were incubated overnight at 4°C. After washing, the protein homogenates in the microtiter wells were incubated with DNP Derivation Solution (200 μL/well) in the dark for 45 min at room temperature. Removal of DNP solution, incubation with 1x blocking buffer (250 μL/well) for 2 h at room temperature, and subsequent washing steps were performed by following the suggested protocol. Each sample was then incubated for 1 h with anti-DNP antibody at room temperature with constant shaking. The color reagent 3,3’,5,5’-tetramethylbenzidine solution was then added to aid color development, according to the manufacturer’s instructions. The absorbance of each homogenate was then read at 450 nm. A standard curve was also prepared with known amounts of protein to calculate the nmol carbonyls/mg protein.

### 2.4 Immunoprecipitation and Immunoblot Analysis

For immunoprecipitation, cytosolic (400 µg/sample) or mitochondrial (500 µg/sample) proteins were incubated with 2 μg of anti-p38K (for cytosolic proteins) or anti-Bax 2D2 (for mitochondrial proteins) overnight at 4°C with constant head-to-tail rotation by the method described [16, 20]. Protein A/G-agarose beads were then added for incubation for another 3 h at 4°C. The agarose beads and bound protein were washed five times with ice-cold 1X phosphate-buffered saline plus 1% CHAPS. Immunoprecipitated proteins bound to the agarose beads were solubilized in SDS-sample buffer for 30 min at room temperature before they were subjected to SDS-PAGE and transferred onto polyvinylidene difluoride (PVDF) membrane (Millipore, Bedford, MA, USA). The membranes were incubated with the primary antibodies overnight at 4°C and then secondary antibodies for 1 h at room temperature. The images were developed by using a kit for enhanced chemiluminescence, as described in a study [18, 21].

### 2.5 Data Processing and Statistical Analysis

Immunoblot data represent the results from at least three separate analyses; while both the carbonylation assay and immunoprecipitation data represent the results from two separate experiments. Densitometry analysis was performed using the software UN-SCAN-IT gel Version 6.1 [22]. The Student’s *t* test was used for the statistical analysis of data where p<0.05 considered statistically significant. Other methods not specifically described were the same as described [16, 18, 21, 22].

## 3 RESULTS

### 3.1 Increased Oxidative Stress in Alzheimer’s Disease Brains

It has been well-established that elevated oxidative stress plays a key role in the pathogenesis of AD and that AD brain tissue shows increased protein carbonylation as well as oxidative inactivation of Prx (Prx-SO_3_), both of which serve as markers of oxidative stress [11, 23-26]. To directly demonstrate the increased oxidative stress, the levels of carbonylated proteins and oxidized peroxiredoxin (Prx-SO_3_) in the cytoplasms from AD and control brain tissues were determined by ELISA and immunoblot analysis, respectively [26-28]. Fig. (**1A**) reveals a significant increase in the level of carbonylated protein in the four AD specimens compared to that of the three control subjects. These results suggest that the brains of individuals with AD are subject to a greater amount of ROS.

**Fig. (1):**
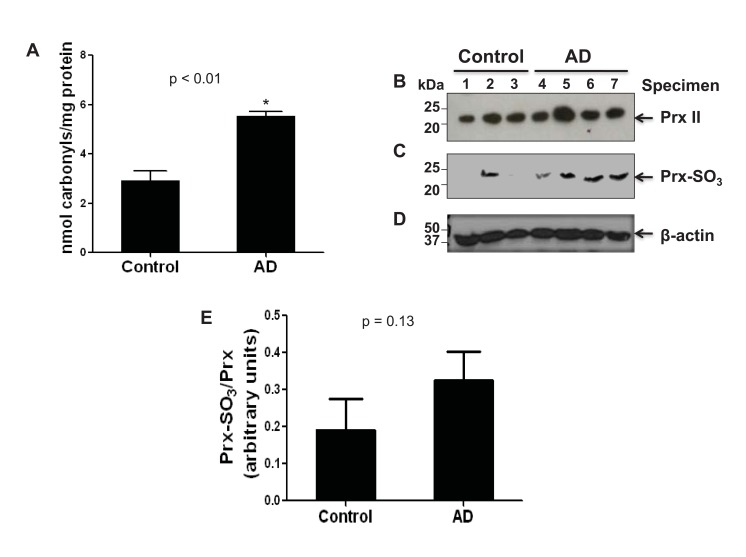
Presence of increased amounts of carbonylated protein, Prx II and its inactive Prx isoform in tissue samples from AD brains. **(A)** The amount of protein carbonylation in cytosolic fractions from the frontal cortex of AD and control brain specimen was measured by ELISA as described in the Method Section. Protein (1 μg) from each of the 7 samples (AD, n=4; control, n=3) was affixed to a 96-well assay plate, and probed for carbonylation via an antibody against DNP. A graph of the amount of carbonyls (nmol carbonyls) per mg of protein is shown. *p<0.01, significantly different from the control samples. **(B-D)** Cytosolic proteins of both AD and control samples (100 μg/well) were separated on 15% SDS-PAGE, transferred to PVDF-Immobilon membranes, and probed with the specific antibodies against Prx II (B), Prx-SO_3_ (C), or β-actin (D), used as a loading control. **(E)** The densitometric quantitation of the immunoblots in B with Prx-SO_3_ normalized to Prx II is presented.

To further elucidate the presence of ROS, an immunoblot analysis of the cytosolic Prx II or Prx-SO_3_ content was performed (Fig. **1B**). Upon the oxidation of Prx, which protects the cell from oxidative stress [29], Prx becomes inactivated. In AD brain specimens as compared to control, there was a marked increase in the amount of the inactive form of Prx, Prx-SO_3_ (Fig. **1C**). This result was further confirmed with additional specimens (consisting of 2 other AD individuals and 2 other control specimens) (data not shown). In contrast, the amount of Prx is similar between four AD and three control brains (Fig. **1B**) as well as 2 additional AD and 2 control brain specimens we evaluated later (data not shown). Immunoblot analysis with the specific antibody against β-actin (Fig. **1D**) is shown as a loading control for protein/specimen. Densitometric analysis, of both shown and not shown immunoblot data (AD, n=6; control, n=5), revealed an approximately 80% increase in the amount of oxidized to non-oxidized Prx in AD compared to control specimens (Fig. **1E**). These data suggest that there is an increase in Prx inactivation in AD due to increased oxidative stress [11, 23-28].

### 3.2 Activation of p38 MAP Kinase in Alzheimer’s Disease Brains

**Fig. (2):**
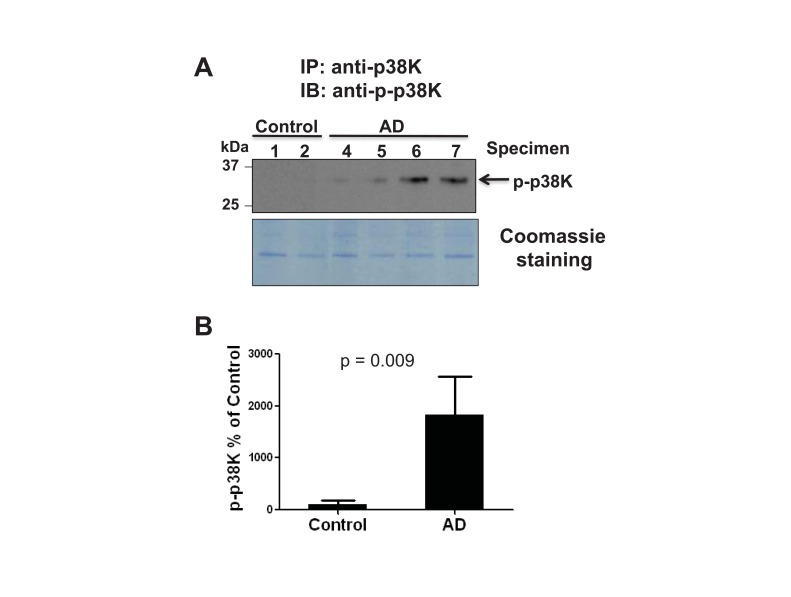
Activation of p38K in AD individuals. **(A)** To accurately determine the activation status of phospho-p38K, brain cytosolic proteins (500 μg/analysis) from AD and control individuals were used for immunoprecipitation (IP) with the specific antibody against p38K. Immunoprecipitated proteins were separated on 12% SDS-PAGE gel, transferred to PVDF-Immobilon, and subjected to IB analysis using an antibody against phospho-p38K (top panel) or Coomassie blue stained (bottom panel). Longer exposure of the same blot did not show any phospho-p38K band in control subjects. **(B)** Densitometric analysis of the immunoblot shows a marked increase in phospho-p38K in AD individuals. P=0.009.

The MAPK family members have all been reported as downstream targets of oxidative stress [10, 16, 27, 28]. In addition, Zhu *et al* reported that the increased level of active MKK6, an upstream kinase of p38K, was detected in AD individuals, suggesting that p38K could be activated (phosphorylated) in AD [19]. Furthermore, cytosolic Prx can efficiently prevent the activation of p38K [30]. These reports and our data of an increased level of inactivated Prx [(*i.e*., Prx-SO_3_ shown in Fig. (**1**)] strongly suggest the possible activation p38K in AD. Based on the increased activation of MKK6 [19], an upstream kinase of p38K, in AD samples compared to control, we evaluated the levels of p38K in the current study. Immunoblot analysis of p38K compared to β-actin used as a loading control (data not shown) revealed that the amount of p38K remained statistically unchanged between AD and control tissues. Given that the level of phospho-p38K was not accurately determined by our immunoblot analysis, p38K phosphorylation was evaluated by immunoprecipitation followed by immunoblot analysis with the specific antibodies against phospho-p38K (Fig. **2A**, top panel). With the Coomassie blue (bottom panel) showing equal protein loading following the immunoprecipitation procedure, only AD samples showed the presence of phosphorylated, active p38K, consistent with the activated MKK6 results as reported [19] (Fig. **2A**). Densitometric analysis of phospho-p38K (Fig. **2A**, top panel) normalized to the Coomassie-stained proteins (Fig. (**2A**), bottom panel) further confirms this strong increase in phospho-p38K in AD compared to control individuals (Fig. **2B**).

### 3.3 Phosphorylation of Bax as a Down-Stream Target of p38k

**Fig. (3):**
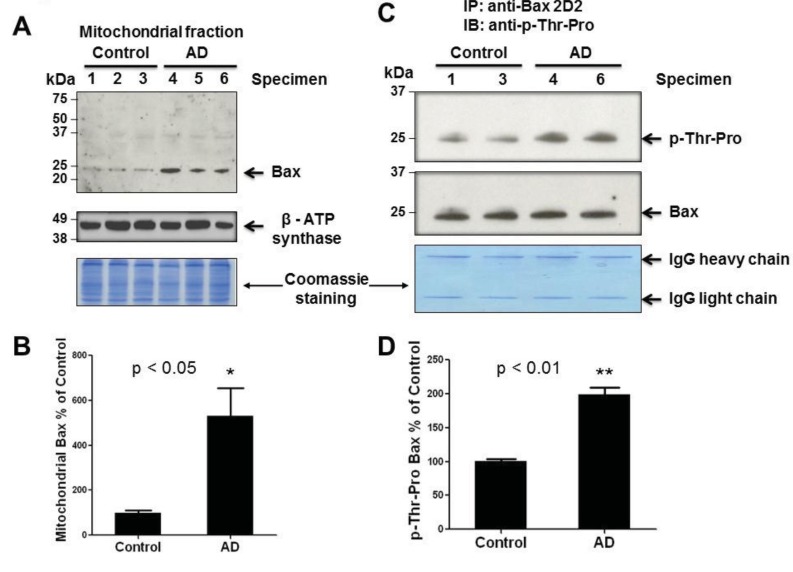
An increase in mitochondrial Bax and phosphorylated Bax. **(A)** Equal amounts of proteins (100 μg/well) from the crude mitochondrial fractions from AD and control individuals were separated on 15% SDS-PAGE, transferred to PVDF-Immobilon, and subjected to immunoblot analyses for Bax (top panel) or ATP synthase-β subunit (middle) and Coomassie blue stained (bottom). **(B)** Densitometric quantitation of mitochondrial Bax in AD patients relative to control subjects is presented. P<0.05. **(C)** Equal amounts of mitochondrial proteins from the two randomly-chosen specimens per group (400 μg protein/analysis) were incubated with 2 ug of anti-Bax antibody (2D2 or 6A7) to immunoprecipitate the mitochondrial Bax protein. Immunoprecipitated Bax proteins were then subjected to IB analysis using a specific antibody against phospho-Thr-Pro (top panel) or Bax (middle). The remaining gel was stained with Coomassie blue (bottom panel) to demonstrate equal protein loading. **(D)** Densitometric quantitation of p-Thr-Pro-Bax in AD patients compared to control subjects is shown. P<0.01.

We previously reported that stress-activated p38K can phosphorylate Thr^167^ of Bax, leading to its translocation to mitochondria and initiation of mitochondria-dependent apoptosis when cultured hepatoma cells are exposed to cell death stimulants such as staurosporine, etoposide, and hydrogen peroxide, or UV exposure [16]. However, it is unknown whether Bax is also phosphorylated and associated with mitochondria in AD compared to controls. Structural analysis revealed that the Thr^167^ residue of Bax is also conserved in many species including humans [protein data base accession number of human Bax: NP_620116]. Therefore, we investigated whether Bax can be a possible downstream target of activated p38K in AD individuals. Under the conditions of similar protein loading, we did not observe significant changes in the amounts of Bax, Bim_EL_, or Bcl-2 in the cytoplasm of AD and control brain specimens (data not shown). However, immunoblot data (top panel) showed a significantly increased amount of Bax (~23 kDa without smaller, degraded products) in cytoplasm (data not shown) or mitochondria of AD brain tissues compared to controls (Fig. **3A**, bottom panel), suggesting that Bax likely plays a role in mitochondria-dependent apoptosis of neuronal cells in AD individuals, as similar to the cultured cells [16]. The densitometric analysis of mitochondria-associated Bax normalized to a mitochondrial marker protein β-subunit of ATP synthase (Fig. **3A**, middle panel) further strengthens these results, revealing a significant increase in the amount of Bax associated with mitochondria in AD individuals compared to that of control subjects (Fig. **3B**).

To further evaluate Bax as a target of p38K, which is a proline-directed Ser/Thr protein kinase, we studied whether Thr residue(s) of Bax was phosphorylated in AD brain tissues, as similar to that observed in cultured hepatoma cells [16]. To demonstrate phosphorylation of Bax in intact brain tissues, Bax in the crude mitochondrial fractions from two randomly-chosen specimens of AD and control was immunoprecipitated by using the specific antibody against Bax. Immunoprecipitated Bax was subjected to immunoblot analysis with the specific anti-phospho-Thr-Pro antibody and anti-Bax 2D2 antibody, probing for phosphorylation of Thr-Pro residue(s) and Bax protein, respectively. Immunoblot and densitometric analyses revealed a significant increase [(approximately 100% in Fig. **3D**)] in the amount of phosphorylated Bax at Thr residue(s) in AD individuals (Figs. **3C** and **D**). In contrast, lower levels of immunoreactive phospho-Thr-Pro bands were detected in control subjects. In addition, little or no immunoreactive phospho-Serine bands in AD and control individuals were observed with a specific anti-phospho-Ser-Pro antibody in our immunoblots, although this antibody recognized Ser residues of another phosphorylated protein (i.e., mitochondrial aldehyde dehydrogenase-2 [31]). The increased amount of phosphorylated Bax on Thr residue(s) in mitochondria suggests elevated levels of mitochondria-dependent apoptosis, which is a possible mechanism of neurodegeneration in AD.

## 4 DISCUSSION

Increased oxidative stress plays a major role in AD and other neurological disorders because the brain is especially vulnerable to reactive oxygen radicals [10, 23-28, 32]. These radicals cause an accumulation of oxidized and carbonylated proteins, which are produced by the ROS and potent peroxynitrite – the product of superoxide and nitric oxide [10, 27]. Peroxinitrite (ONOO^-^) has been proven to be abundant and widespread throughout the brain of individuals with AD [33, 34].

Prx acts as a protective mechanism for the cell against oxidative stress in neuronal tissues [26, 29, 30, 35]. The active form of Prx may be up-regulated in AD brains in response to the increase of ROS common among AD individuals. Many reports also assert that the inactivation of Prx provides a link between oxidative stress and neurodegeneration [26, 29]. However, we did not observe any increment in the amount of Prx while the oxidized, inactive Prx-SO_3_ content was increased in all 4 AD specimens and one control individual used in our study Fig. (**1C**). We do not know the reason for the elevated levels of oxidized Prx in this particular control individual. Nonetheless, the Prx-SO_3_ data, along with the protein carbonylation results (Fig. **1**), provide evidence for a significantly increased level of oxidative stress in AD compared to control used in the current study.

**Fig. (4):**
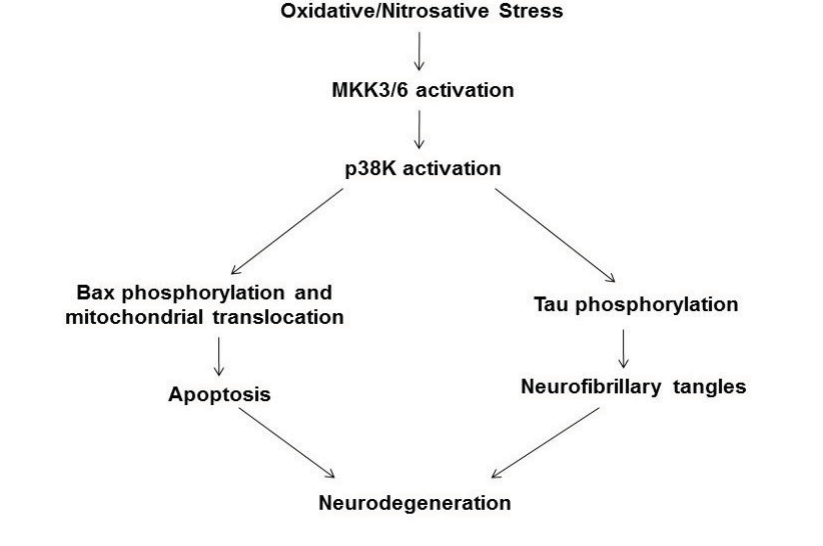
A proposed model of AD neurodegeneration through the stress-activated p38K and apoptosis. Oxidative stress induces the activation of p38K and its upstream effector, MKK6. Activated (phosphorylated) p38K will then phosphorylate Bax in the cytoplasm, causing its translocation to mitochondria to initiate the mitochondria-dependent apoptosis process, and then ultimately neurodegeneration. In addition, activated p-P38K can also phosphorylate Tau protein, causing aggregations called neurofibrillary tangles, which also contribute to neurodegeneration. This model is similar to those previously reported [43-45].

In this study, we elucidated one probable mechanism of neuronal cell death and neurodegeneration (Fig. **4**) by studying the role of the stress activated p38K pathway in phosphorylation of Bax and its association in mitochondria. Markedly elevated levels of ROS have been shown to activate the MAPK pathway, ultimately leading the cell towards apoptosis [8, 10, 16, 27, 28]. In cultured cells, oxidative inactivation of Prx contributes to activation p38K [30]. Furthermore, the p38K pathway, in particular, has been identified as being dysregulated, through the activation of MKK6, reported only in AD samples but not age-matched controls, and therefore playing an active role in the pathogenesis of AD [19].

Knowing that p38K is activated in AD (Fig. **2A**) and that the p38K pathway leads to apoptosis [16], we sought to investigate the possible mechanism of neurodegeneration in AD. Activated p38K can phosphorylate downstream targets such as pro- and anti-apoptotic members of the Bcl-2 family proteins: Bim_EL_ [36], Bcl-2 [6], Bax [16], etc. Phosphorylation of one of the three Bcl-2 family members likely leads to apoptosis. However, little changes in the amount of Bim_EL_ and Bcl-2 (both non-phosphorylated and phosphorylated proteins) between AD individuals and control subjects indicate that Bax could become the major downstream target of p38K in this *in vivo* study of AD specimens.

Previous research revealed that there are three mechanisms through which pro-apoptotic Bax can induce mitochondria-mediated cell death. One mechanism is through the cleavage of Bax by calpain, a cysteine protease [37]. Through this mechanism, Bax is cleaved at mitochondria during apoptosis into a p18/Bax molecule that is more potent than the uncleaved p21/Bax [37-39]. However, Bax cleavage can be ruled out as a possible mechanism of apoptosis based on the fact that no cleavage product of Bax was recognized in either cytosolic or mitochondrial fractions of AD or control tissues by immunoblot analysis. A second mechanism of Bax-mediated apoptosis involves a pathway in which the ratio of Bax/Bcl-2 increases through the upregulation of Bax and subsequent down-regulation of Bcl-2 [40]. Based on little changes in the amounts of Bcl-2 and Bax in the cytoplasm of AD and control specimens, this mechanism, through altering the Bax/Bcl-2 ratio, is unlikely to be the source of apoptosis in this *in vivo* study of AD. The third possible mechanism could be translocation of Bax to the mitochondria to initiate mitochondrial permeability change and release of mitochondrial cytochrome c prior to apoptosis, as demonstrated in many different types of cultured cells [16, 41]. We previously reported that p38K can phosphorylate Bax at Thr^167^ in cultured hepatoma cells [16]. However, it is unknown whether the results observed in cultured cells can be replicated in *in vivo* tissue specimens. Therefore, the main goal of this study was to explore the role of p38K in phosphorylating Bax and the subsequent translocation of phosporylated Bax to mitochondria in AD specimens. Our current data clearly support this mechanism of apoptosis with increased translocation of phosphorylated Bax to mitochondria, in AD neurons compared to control, suggesting the Bax-mediated mitochondria-dependent apoptosis in AD. Our data also reveals that the significant increase in mitochondrial Bax was actually phosphorylated at one or more threonine residue(s). The results of Bax phosphorylation and increased association with mitochondria (Fig. **3**) confirm many earlier reports conducted with cultured cells [16, 41]. To our knowledge our data provides the first evidence of a possible mechanism of p38K-Bax-mediated mitochondria-dependent apoptosis in AD.

## CONCLUSION

In conclusion, we propose a probable mechanism by which elevated oxidative stress leads to neurodegeneration via apoptosis and neurofibrillary tangles (Fig. **4**). Oxidative stress, as evidenced through the increases in protein carbonylation and Prx oxidation, activates p38K which can then proceed in two ways. The first is through the phosphorylation of Bax at Thr residue(s) and subsequent translocation of phosphorylated Bax to mitochondria to cause mitochondria-dependent apoptosis of neuronal cells [16, 42]. In addition, a second pathway is that p-p38K can phosphorylate tau, causing its accumulation as neurofibrillary tangles in AD neurons, as documented [43-45]. Both downstream targets of activated p-p38K likely lead to the neurodegeneration associated with AD.

## Figures and Tables

**Table 1:** Demographic data for specimen used in this study. Adapted from the report by Zhu *et al.* [19]. PMI, post-mortem interval.

**Specimen**	**ID Number**	**Diagnosis**	**Age**	**PMI(hrs)**
**1**	**A86-402**	**Control**	**64**	**3**
**2**	**A89-076**	**Control**	**75**	**17**
**3**	**A90-193**	**Control**	**68**	**Not available**
**4**	**A89-188**	**AD**	**72**	**4**
**5**	**A90-010**	**AD/Lewy Bodies**	**72**	**3**
**6**	**A92-404**	**AD**	**69**	**6.5**
**7**	**A96-076**	**AD**	**78**	**3**
**Additional Specimen**				
**8**	**A91-473**	**Control**	**76**	**6**
**9**	**O98-010**	**Control**	**NA**	**NA**
**10**	**A92-218**	**AD**	**80**	**4.5**
**11**	**A99-109**	**AD**	**87**	**3**

## LIST OF ABBREVIATIONS

AD: Alzheimer’s Disease
Prx: Peroxiredoxin
Prx-SO_3_: Oxidized Inactive Prx
ROS: Reactive Oxygen Species
MAPK: Mitogen-Activated Protein Kinase
